# Learning the protein language of proteome-wide protein-protein binding sites via explainable ensemble deep learning

**DOI:** 10.1038/s42003-023-04462-5

**Published:** 2023-01-19

**Authors:** Zilong Hou, Yuning Yang, Zhiqiang Ma, Ka-chun Wong, Xiangtao Li

**Affiliations:** 1 School of Artificial Intelligence, Jilin University, Jilin, China; 2 Information Science and Technology, Northeast Normal University, Jilin, China; 3 Department of Computer Science, City University of Hong Kong, Hong Kong SAR, China

**Keywords:** Protein function predictions, Computational models

## Abstract

Protein-protein interactions (PPIs) govern cellular pathways and processes, by significantly influencing the functional expression of proteins. Therefore, accurate identification of protein-protein interaction binding sites has become a key step in the functional analysis of proteins. However, since most computational methods are designed based on biological features, there are no available protein language models to directly encode amino acid sequences into distributed vector representations to model their characteristics for protein-protein binding events. Moreover, the number of experimentally detected protein interaction sites is much smaller than that of protein-protein interactions or protein sites in protein complexes, resulting in unbalanced data sets that leave room for improvement in their performance. To address these problems, we develop an ensemble deep learning model (EDLM)-based protein-protein interaction (PPI) site identification method (EDLMPPI). Evaluation results show that EDLMPPI outperforms state-of-the-art techniques including several PPI site prediction models on three widely-used benchmark datasets including Dset_448, Dset_72, and Dset_164, which demonstrated that EDLMPPI is superior to those PPI site prediction models by nearly 10% in terms of average precision. In addition, the biological and interpretable analyses provide new insights into protein binding site identification and characterization mechanisms from different perspectives. The EDLMPPI webserver is available at http://www.edlmppi.top:5002/.

## Abstract (web summary)

An ensemble deep learning model (EDLM)-based protein-protein interaction (PPI) site identification method (EDLMPPI) accurately predicts PPIs through protein language models and outperforms state-of-the-art methods on multiple benchmark sets.

## Introduction

Protein–protein interactions (PPIs) have an essential role in all the major cellular processes which assist in elucidating protein function, but also for interpreting most of the biology of the cells. In particular, key proteins in these protein interactions may provide the basis for the development of targeted therapeutic drugs in the related diseases, also informing on the underlying molecular basis of diseases^1^. While there are numerous databases such as BioLip^2^ and PDB^3^ available for querying protein–protein interaction sites, they appear overwhelming due to the increasing number of proteins now known to humans^4^. Similarly, biological experiments for the detection of binding sites, such as two-hybrid analysis and affinity systems, are very time-consuming and expensive^5^. To bridge this gap, many computational methods have been developed to address protein interactions and associated sites. In recent years, many deep learning-based protein interaction site identification models have been proposed by incorporating the powerful feature extraction capabilities of deep learning, resulting in a qualitative leap in prediction performance compared to traditional machine learning. For example, Zeng et al.^6^ used TextCNN as a feature extractor to learn features using convolutional kernels of different sizes, which can improve the prediction performance. Xie et al.^7^ adopted a simple CNN to learn local features between residues. Yang et al.^8^ presented a deep neural network with local weight sharing to predict amino acid interaction sites. Sun et al.^9^ developed a deep learning architecture based on residual neural networks for predicting interacting amino acids in transmembrane proteins. Zhang et al.^10^ used a simplified LSTM to predict PPI, aiming to learn the contextual information of the features using LSTM’s ability to grasp the global context. Li et al.^11^ integrated local contextual information and long-range dependencies by incorporating CNN and RNN, which improves the model’s performance. Unfortunately, most of these computational methods are very unstable and poorly generalized, especially for these highly unbalanced benchmark datasets, implying some room for improvement.

On the other hand, a plethora of protein sequence encoding methods has been proposed for modeling protein sequences into a feature matrix. One-hot encoding of protein interaction sites is a very efficient method that has been used in many computational approaches^10,12^. However, they cannot accurately express functional differences between amino acids. Position-specific scoring matrix (PSSM) is frequently employed for sequence-level and residue-level prediction tasks to characterize the relationship between sequences and functions^4,6,10,11,13^, which is relatively time-consuming due to the fact that PSSM requires sequence alignment of large databases. Recently, the development of word embedding models in natural language processing has provided the possibility of addressing protein-coding. Some word embedding models such as Word2Vec^14^, Doc2Vec^15^, fastText^16^, and GloVe^17^ have been widely adopted in the field of bioinformatics; for instance, Zeng et al.^6^ encoded amino acids using a static word embedding model based on ProtVec^18^, which improves the accuracy of PPIs prediction. The iCircRBP-DHN proposed by Yang et al.^19^ advances the identification accuracy of circRNA-RBP interaction sites by Doc2Vec^15^. Min et al.^20^ carried out chromatin accessibility prediction by using GloVe^17^ as an embedding method for gene sequences. Hamid^21^ used Word2Vec^22^ to represent protein sequences for differentiating bacteriocins. Unfortunately, such static word vector embeddings do not capture well the association between sequences and structures and neglect the potential connections between sequence contexts. To address these limitations, dynamic word embeddings, as represented by the Bidirectional Encoder Representations from Transformers (BERT) model have demonstrated very good performance in semantic analysis, able to learn sequence context of protein sequences by pre-training large-scale unlabeled corpora in a bidirectional manner^23–25^.

**Fig. 1:**
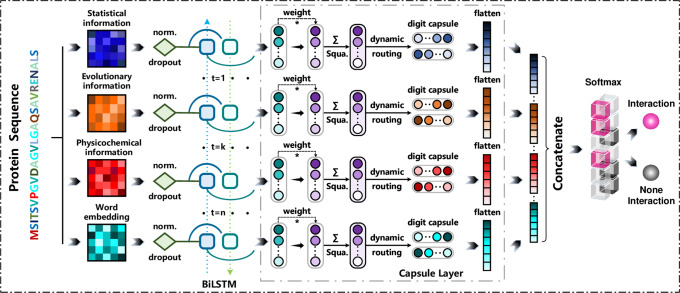
Overview of the proposed method, an ensemble deep learning model (EDLMPPI)-based protein–protein interaction site identifier consisting of two main components: Bi-directional Long Short-Term Memory (BiLSTM) for extracting long-range dependencies of features and capsule network for exploring the intrinsic association between features and preserving inter-sample location information. On the one hand, this design can capture the correlation between features in both directions and fully considers the contextual information. On the other hand, the capsule can retain key information as much as possible while reducing the dimensionality of features, avoiding information leakage, and improving the efficiency of the algorithm.

In our study, we propose an ensemble deep learning model (EDLMPPI)-based protein–protein interaction site identification method, as depicted in Fig. 1. We suggest adopting ProtT5 based on transformer architecture as the amino acid feature extractor, to fully exploit the global contextual association of each amino acid, and then, we incorporate eleven additional feature descriptors to further enrich the feature representation. In EDLMPPI, the deep learning architecture is composed of BiLSTM^26^ and capsule network^27^, where BiLSTM can learn features in both forward and backward directions of protein sequences in a comprehensive manner, and the capsule network can further discover correlations between features. To cope with the impact of the unbalanced datasets, we train multiple deep learning models to form ensemble deep learning and then perform predictions. To investigate the effectiveness of our proposed EDLMPPI, we conducted experiments on the network mechanism and feature extraction parts. All experiments were based on the training and test sets described in the section “Methods”. The validation set was randomly token as 20% of the training set, and we also used stratified random sampling to divide the validation set to ensure consistency of the distribution of the training and validation sets. To validate the effectiveness of EDLMPPI, we compare it with ten different machine learning models and deep learning models on the benchmark datasets. Further, we also compare EDLMPPI with other PPI site prediction models and demonstrated that EDLMPPI is in front by a large margin, which validates the efficiency of EDLMPPI’s feature extraction and network architecture. To explore the biological significance of EDLMPPI, we extract the structural domains of protein sequences. Compared with other methods, the interaction sites predicted by EDLMPPI showed a higher correlation with the native sites in the structural domain. In addition, we conducted an interpretable analysis to demonstrate the internal process of EDLMPPI’s feature representation. We built a web server for EDLMPPI prediction at http://www.edlmppi.top:5002/.

## Results and discussion

### EDLMPPI can provide a more efficient scheme for characterizing protein sequences

**Fig. 2:**
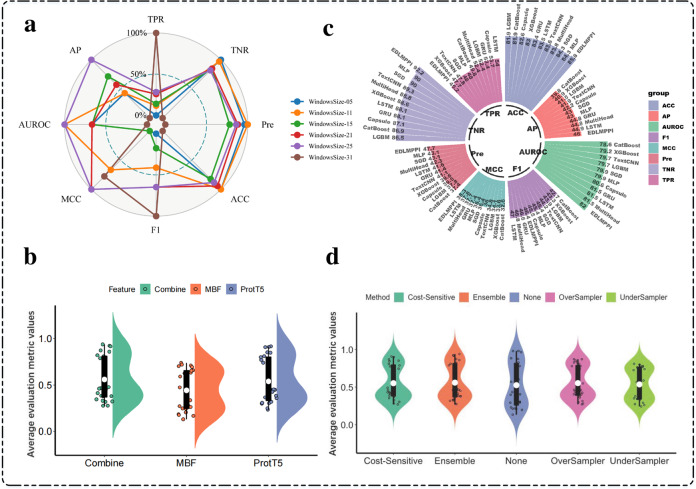
Experimental results are presented to reveal the effectiveness of the model. **a** Radar chart of evaluation indicators corresponding to the different window sizes. **b** Showing the performance comparison of ProtT5, MBF, and combined features on the classifier, where the “Average evaluation metric values” refers to the average of the eight evaluation metrics (including TPR, TNR, Pre, ACC, F1, MCC, AUROC, and AP) for the different feature descriptors on these three datasets. **c** Demonstrating the performance comparison between the EDLMPPI architecture and 10 mainstream machine learning models and deep learning models: EDLMPPI is particularly strong in key metrics. **d** Performance comparison between different methods for imbalance dataset resolution, where the “Average evaluation metric values” refers to the average of the eight evaluation metrics (including TPR, TNR, Pre, ACC, F1, MCC, AUROC, and AP) for the different algorithms on these three datasets.

In our study, we adopted a multi-channel strategy to form combined features with MBF (Multi-source Biological Features, including the evolutionary information, physical properties, and physicochemical properties of protein residues) and ProtT5 as inputs to the model, respectively. Then, the two sets of vectors were concatenated and normalized before the softmax classification layer. In MBF, the sliding window mechanism was employed to encode the local contextual information for each residue, which can effectively prevent overfitting and improve the generalization of the model. Moreover, for a window size of *n* (*n* is an odd number), the middle-most amino acid is the target amino acid to be predicted, and the sliding step is 1. Therefore, we first conducted an experiment to find the optimal window size in MBF by evaluating the performance of the MBF model with different window sizes from the set {5, 11, 15, 21, 25, 33}. The experimental results of different window sizes are summarized in Fig. 2a with Dset_448 as an example. It is clear that the model achieved the best performance measured by several key metrics including AP, AUROC, and MCC for a window size of 25. However, the overall performance of the algorithm decreased with a window size of 31, which indicates that larger windows are not always better. Therefore, in our study, we choose a window size of 25 as the final size.

In addition, to further investigate the superiority of our proposed feature descriptor, we compared the combined features in EDLMPPI with a single feature descriptor including MBF and ProtT5, respectively. The experimental results are tabulated in Table 1 and Fig. 2b. It can be observed that combining the features of MBF and ProtT5 greatly outperformed the individual feature descriptors on all three datasets. Indeed, for the evaluation metric AP, frequently used to evaluate unbalanced data, the combined features surpassed MBF on the three datasets, respectively, and outperformed ProtT5 by 1.8%, 3%, and 2.9%, respectively, revealing that the combined features enriched the protein expression and enhanced the performance of the model. Moreover, when comparing Prot5 and MBF, it can also be revealed that the AP values of Prot5 perform better than on those three datasets and outperforms MBF by 10.7%, 11.2%, and 8.6%, respectively for AUROC, unveiling the effectiveness of dynamic word embedding in protein–protein binding site prediction. The reason may be that ProtT5 captured better the difference between amino acids (binding sites and non-binding sites) from our labeled training data while MBF had difficulty distinguishing amino acid specificity based on evolutionary information and other biological functions.

**Table 1:** Performance comparison under different feature descriptors.

	TPR	TNR	Pre	ACC	F1	MCC	AUROC	AP
*Dset_448*								
MBF	0.537	0.738	0.243	0.710	0.335	0.205	0.703	0.272
ProtT5	0.448	0.916	0.455	0.852	0.451	0.366	0.810	0.442
Combine	0.452	0.922	0.477	0.858	0.464	0.383	0.820	0.460
*Dset_72*								
MBF	0.474	0.720	0.167	0.694	0.247	0.130	0.658	0.185
ProtT5	0.336	0.908	0.303	0.848	0.319	0.234	0.770	0.300
Combine	0.318	0.938	0.377	0.872	0.345	0.276	0.788	0.330
*Dset_164*								
MBF	0.591	0.634	0.263	0.626	0.364	0.176	0.654	0.283
ProtT5	0.308	0.909	0.427	0.800	0.358	0.248	0.740	0.384
Combine	0.323	0.916	0.460	0.809	0.380	0.277	0.755	0.413

### Comparing ProtT5 with other protein language models

In recent years, language models based on Transformer architecture have been widely used in protein prediction problems. The self-attention-based Transformer can directly calculate the two-by-two association between residues and capture the interdependence between amino acids at different positions. In addition to ProtT5, several alternative protein pre-training models including ESM-1b^28^ and ProGen2^29^ have been proposed to characterize protein sequences. ESM-1b uses a RoBERTa-based architecture with the Uniref50 2018_03 database as the unsupervised training corpus while using pre-activation layer normalization to optimize hyperparameters in the translator. ProGen2 was scaled to 6.4 billion parameters and trained on different sequence datasets with more than 1 billion proteins from genomic, metagenomic, and immune repertoire databases. For a fair comparison, we replaced the embedding representation learned by ProtT5 with the embedding representation learned by ESM-1b and ProGen2. The experimental results are summarized in Table 2. As depicted in this table, we observe that ProtT5 is superior to ESM-1b and ProGen2 in AP and AUROC, demonstrating that the ProtT5 is more suited for characterizing the amino acid sequences for protein–protein binding events.

**Table 2:** Performance comparison under different protein language models.

	TPR	TNR	Pre	ACC	F1	MCC	AUROC	AP
*Dset_448*								
ESM-1b	0.725	0.652	0.246	0.662	0.368	0.264	0.759	0.349
ProGen2	0.714	0.602	0.220	0.617	0.336	0.218	0.715	0.287
ProtT5	0.448	0.916	0.455	0.852	0.451	0.366	0.810	0.442
*Dset_72*								
ESM-1b	0.674	0.678	0.199	0.678	0.307	0.226	0.738	0.253
ProGen2	0.684	0.554	0.154	0.568	0.251	0.147	0.658	0.176
ProtT5	0.336	0.908	0.303	0.848	0.319	0.234	0.770	0.300
*Dset_164*								
ESM-1b	0.732	0.542	0.261	0.576	0.375	0.211	0.690	0.324
ProGen2	0.758	0.436	0.229	0.495	0.352	0.153	0.650	0.288
ProtT5	0.308	0.909	0.427	0.800	0.358	0.248	0.740	0.384

### EDLMPPI can effectively deal with the overfitting problem caused by data imbalance

As the number of residues in the binding sites is only one-tenth of the total number, this unbalanced data pushes the model training to focus on the major class and ignores the minor class, leading to overfitting of the model^30–32^. To address this issue, we proposed employing ensemble deep learning to tackle the skewed distribution of categories of unbalanced datasets. To investigate the performance of the ensemble model, we compared it to three other different unbalanced data processing algorithms, including cost-sensitive model^33^, random over-sampling^34^, and random under-sampling^34^ under these three datasets. In detail, the cost-sensitive model^33^ focuses on the samples of categories by optimizing the lowest total cost of classification errors. Over-sampling^34^ generates new samples for the underrepresented classes by random sampling, while under-sampling^34^ randomly removes redundant samples from the major class sample.

The experimental results are summarized in Table 3 and Fig. 2d. Generally, the ensemble model performed the best, obtaining higher MCC, AUROC, and AP scores. In terms of AP scores on the three datasets, the ensemble learning algorithm comparatively outperformed the competing algorithms with 46.0%, 33.0%, and 41.3%, respectively, indicating an improved generalization performance with the asymmetric bagging method. In addition, the average precision of the over-sampling method on the three data sets was 43.9%, 31.5%, and 40.4%, respectively, which was lower than the ensemble learning method since the over-sampling method destroys the dependencies between features and limits the ability of the model to find correlations between features. It is worth noting that the under-sampling method can be considered as a sub-model of the ensemble deep learning model, which lags for AUROC and AP scores by 1.1% to 3.9% on all three datasets compared to the ensemble learning method.

**Table 3:** Comparison of algorithm performance with different unbalanced dataset processing strategies.

	TPR	TNR	Pre	ACC	F1	MCC	AUROC	AP
*Dset_448*								
None	0.197	0.979	0.601	0.873	0.297	0.293	0.807	0.433
Cost-Sensitive	0.491	0.898	0.431	0.843	0.459	0.369	0.809	0.430
Ensemble	0.452	0.922	0.477	0.858	0.464	0.383	0.820	0.460
UnderSampler	0.652	0.798	0.336	0.778	0.444	0.350	0.809	0.435
OverSampler	0.549	0.870	0.398	0.826	0.462	0.381	0.807	0.439
*Dset_72*								
None	0.209	0.963	0.398	0.883	0.274	0.230	0.779	0.305
Cost-Sensitive	0.386	0.905	0.325	0.850	0.353	0.270	0.775	0.304
Ensemble	0.318	0.938	0.377	0.872	0.345	0.276	0.788	0.330
UnderSampler	0.587	0.783	0.243	0.762	0.343	0.261	0.769	0.291
OverSampler	0.464	0.870	0.298	0.827	0.363	0.271	0.779	0.315
*Dset_164*								
None	0.134	0.978	0.570	0.825	0.217	0.213	0.741	0.404
Cost-Sensitive	0.418	0.868	0.412	0.787	0.415	0.284	0.744	0.404
Ensemble	0.323	0.916	0.460	0.809	0.380	0.277	0.755	0.413
UnderSampler	0.565	0.761	0.343	0.725	0.427	0.274	0.738	0.378
OverSampler	0.452	0.850	0.401	0.778	0.425	0.281	0.742	0.404

In summary, we can conclude that the ensemble deep learning method based on asymmetric bagging assures the efficiency of algorithm execution and enhances its identification performance, by comparatively reducing the impact of the unbalanced data sets.

### Comparing EDLMPPI with different machine learning algorithms

To study the effectiveness of EDLMPPI, we compared it with five machine learning methods, including three ensemble learning methods (XGBoost^35^, LightGBM^36^, and CatBoost^37^) and two other machine learning methods, SGDClassifier (Stochastic Gradient Descent), and MLPClassifier (Multi-Layer Perception). Figure 2c and Table 4 depict the experimental results of the different algorithms on all three datasets. From the results, we see that our proposed model had better performance than the five other machine learning algorithms on all three datasets. In particular, on Dset_448, EDLMPPI outperformed the machine learning methods by 2.1–3.4% in the average AUROC and by 3.0–6.2% for the average AP on the three datasets, indicating the large improvement in the predictive ability of EDLMPPI. Moreover, since the same feature descriptor is adopted by EDLMPPI and these machine learning algorithms, we observe from the results that the comprehensive performance of the deep learning method was stronger than that of traditional machine learning, indicating that the deep learning method can explore the potential connection between protein sequence and structure better, thereby improving the prediction of protein binding sites performance, which further proves the effectiveness of EDLMPPI.

**Table 4:** Performance of different machine learning methods and deep learning methods on Dset_448, Dset_72, and Dset_164.

	TPR	TNR	Pre	ACC	F1	MCC	AUROC	AP
*Dset_448*								
XGBoost	0.477	0.886	0.396	0.830	0.433	0.336	0.792	0.409
LGBM	0.524	0.865	0.378	0.819	0.439	0.341	0.797	0.415
CatBoost	0.498	0.869	0.374	0.819	0.427	0.326	0.786	0.398
SGD	0.480	0.900	0.431	0.843	0.454	0.364	0.799	0.430
MLP	0.480	0.900	0.431	0.843	0.454	0.364	0.799	0.430
TextCNN	0.478	0.893	0.411	0.836	0.442	0.348	0.797	0.419
Capsule	0.537	0.871	0.395	0.826	0.455	0.360	0.806	0.422
GRU	0.534	0.881	0.414	0.834	0.466	0.374	0.815	0.448
LSTM	0.540	0.881	0.416	0.835	0.470	0.378	0.815	0.449
MultiHead	0.524	0.888	0.423	0.838	0.468	0.377	0.815	0.448
EDLMPPI	0.452	0.922	0.477	0.858	0.464	0.383	0.820	0.460
*Dset_72*								
XGBoost	0.501	0.818	0.246	0.785	0.330	0.239	0.743	0.274
LGBM	0.410	0.880	0.288	0.830	0.338	0.249	0.759	0.295
CatBoost	0.406	0.876	0.279	0.826	0.330	0.240	0.751	0.270
SGD	0.521	0.812	0.247	0.781	0.335	0.246	0.748	0.293
MLP	0.504	0.826	0.256	0.792	0.340	0.250	0.759	0.283
TextCNN	0.418	0.878	0.289	0.829	0.342	0.253	0.760	0.301
Capsule	0.544	0.829	0.274	0.799	0.364	0.282	0.773	0.305
GRU	0.425	0.878	0.293	0.830	0.347	0.259	0.774	0.308
LSTM	0.421	0.881	0.296	0.832	0.347	0.260	0.765	0.316
MultiHead	0.279	0.940	0.354	0.870	0.312	0.244	0.776	0.308
EDLMPPI	0.318	0.938	0.377	0.872	0.345	0.276	0.788	0.330
*Dset_164*								
XGBoost	0.553	0.759	0.336	0.722	0.419	0.263	0.723	0.361
LGBM	0.480	0.821	0.372	0.759	0.419	0.274	0.733	0.375
CatBoost	0.576	0.741	0.330	0.711	0.419	0.263	0.719	0.364
SGD	0.496	0.804	0.359	0.749	0.417	0.268	0.730	0.371
MLP	0.457	0.822	0.362	0.756	0.404	0.255	0.720	0.358
TextCNN	0.412	0.868	0.409	0.786	0.410	0.280	0.728	0.394
Capsule	0.438	0.852	0.395	0.777	0.415	0.279	0.741	0.388
GRU	0.443	0.853	0.399	0.779	0.420	0.285	0.747	0.394
LSTM	0.447	0.855	0.405	0.781	0.425	0.291	0.751	0.401
MultiHead	0.447	0.855	0.405	0.781	0.425	0.291	0.751	0.401
EDLMPPI	0.323	0.916	0.460	0.809	0.380	0.277	0.755	0.413

### Comparing EDLMPPI with different deep learning architectures

To validate the effectiveness and sophistication of the revised architecture of EDLMPPI, we compared it with other five deep learning models including TextCNN^38^, Single-Capsule^27^, BiLSTM^39^, BiGRU^40^, and Multi-Head Attention^41^ using the same feature descriptors. The experimental results of the different deep learning models are depicted in Fig. 2c and Table 4, where we see that EDLMPPI performed comparatively better than the other deep learning models, measured by the evaluation metric AP, outperforming the second-ranked Multi-Head-Attention by 1.2%, 2.2%, and 1.2% on the three datasets, respectively. Moreover, the intuitive view of TextCNN’s performance was weaker than several other deep learning models, which is consistent with our expectation that the CNN structure only extracted the local features, undermining the integrity of Prot5’s context-based embedding. In addition, LSTM and GRU perform comparably on Dset_448 and Dset_72, but LSTM performs better than GRU on Dset 164, which is the reason for choosing LSTM to learn long-term dependencies in the final model EDLMPPI.

### Comparing EDLMPPI with other PPIs prediction methods

**Fig. 3:**
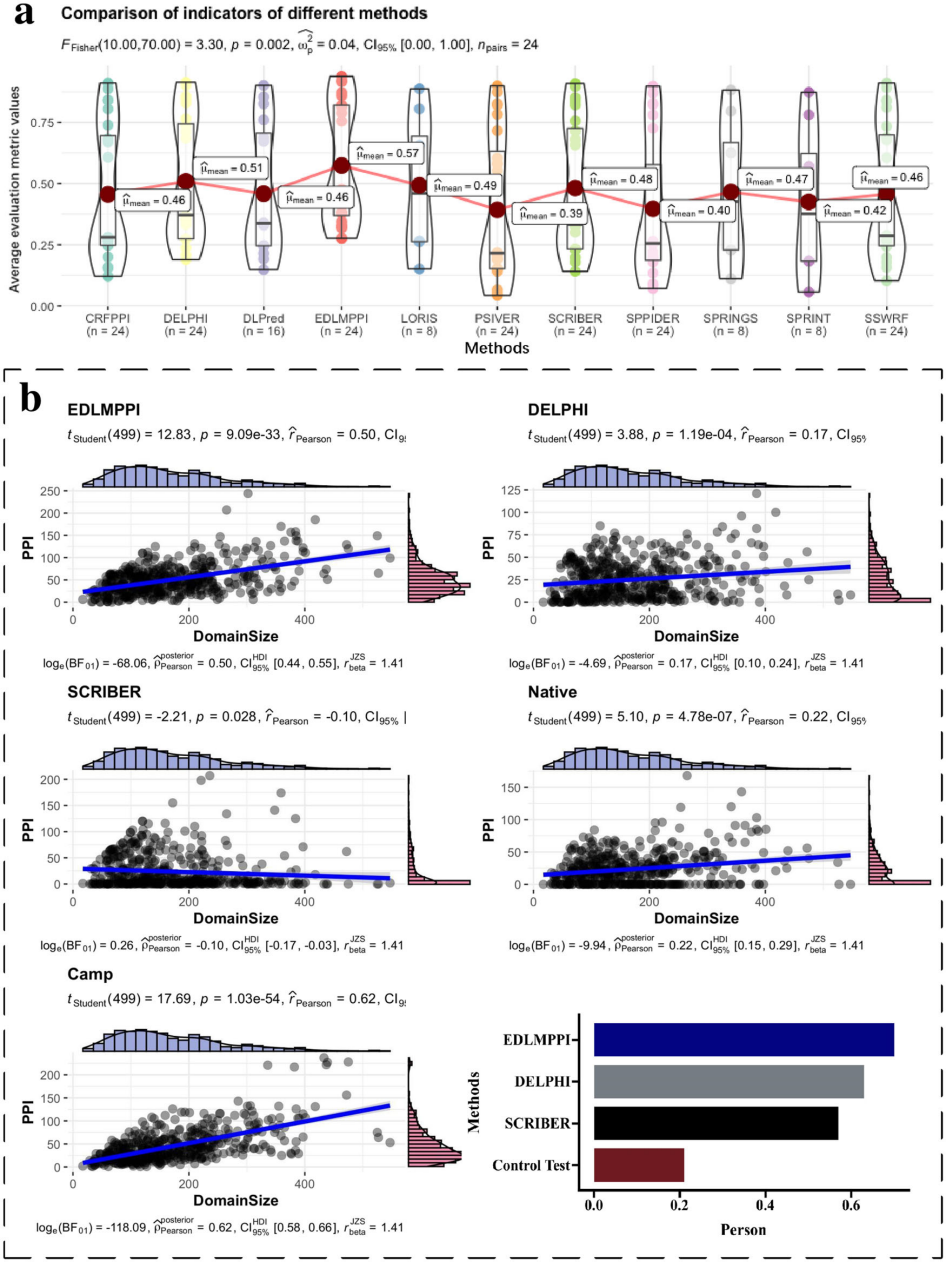
Display of the results of comparative experiments and biological analysis experiments. **a** Demonstrating the results of comparisons between EDLMPPI and ten other competitive methods, with the “Average evaluation metric values” referring to the average of the eight evaluation metrics (including TPR, TNR, Pre, ACC, F1, MCC, AUROC, and AP) for the different methods on these three datasets. **b** A comparison of the predicted PPIs from EDLMPPI, DELPHI, and SCRIBER compared to native PPIs. By calculating the proportion of PPIs in each domain, EDLMPPI and native PPIs have the highest correlation.

To further test the advancement brought by EDLMPPI, we compared it with ten current PPI prediction methods including SPPIDER^42^, SPRINT^43^, PSIVER^44^, SPRINGS^45^, LORIS^46^, CRFPPI^47^, SSWRF^48^, DLPred^49^, SCRIBER^13^, and DELPHI^11^. We obtained the prediction scores for each protein sequence in the test dataset through the web server or the available source codes of these algorithms. We adopted TPR, TNR, Pre, ACC, F1, MCC, AUROC, and AP as the evaluation criteria and MCC, AUROC, and AP as the important determinants for evaluating the merits of the models that are frequently used to evaluate unbalanced data^13^. The prediction results are summarized in Table 5 and Fig. 3a.

**Table 5:** Performance comparison of the different predictors.

	TPR	TNR	Pre	ACC	F1	MCC	AUROC	AP
*Dset_448*								
SPPIDER	0.202	0.870	0.194	0.781	0.198	0.071	0.517	0.159
SPRINT	0.183	0.873	0.183	0.781	0.183	0.057	0.570	0.167
PSIVER	0.191	0.874	0.191	0.783	0.191	0.066	0.581	0.170
SPRINGS	0.229	0.882	0.228	0.796	0.229	0.111	0.625	0.201
LORIS	0.264	0.887	0.263	0.805	0.263	0.151	0.656	0.228
CRFPPI	0.268	0.887	0.264	0.805	0.266	0.154	0.681	0.238
SSWRF	0.288	0.891	0.286	0.811	0.287	0.178	0.687	0.256
SCRIBER	0.334	0.896	0.332	0.821	0.333	0.230	0.715	0.287
DELPHI	0.371	0.901	0.371	0.829	0.371	0.272	0.737	0.337
EDLMPPI	0.452	0.922	0.477	0.858	0.464	0.383	0.820	0.460
*Dset_72*								
SPPIDER	0.188	0.898	0.179	0.823	0.183	0.084	0.522	0.134
PSIVER	0.152	0.899	0.152	0.820	0.152	0.052	0.604	0.141
CRFPPI	0.248	0.911	0.248	0.840	0.248	0.158	0.669	0.200
SSWRF	0.246	0.911	0.246	0.840	0.246	0.157	0.678	0.198
SCRIBER	0.232	0.909	0.232	0.837	0.232	0.141	0.680	0.198
DLPred	0.246	0.901	0.246	0.826	0.246	0.148	0.688	0.215
DELPHI	0.274	0.914	0.274	0.847	0.274	0.189	0.711	0.237
EDLMPPI	0.318	0.938	0.377	0.872	0.345	0.276	0.788	0.330
*Dset_164*								
SPPIDER	0.264	0.828	0.253	0.726	0.258	0.090	0.528	0.220
PSIVER	0.217	0.826	0.216	0.716	0.216	0.043	0.554	0.205
CRFPPI	0.280	0.841	0.280	0.739	0.280	0.121	0.608	0.267
SSWRF	0.266	0.838	0.266	0.734	0.266	0.103	0.606	0.243
SCRIBER	0.327	0.851	0.327	0.756	0.327	0.179	0.657	0.301
DLPred	0.338	0.854	0.338	0.760	0.338	0.192	0.672	0.330
DELPHI	0.352	0.857	0.352	0.765	0.352	0.209	0.685	0.332
EDLMPPI	0.323	0.916	0.460	0.809	0.380	0.277	0.755	0.413

We see that EDLMPPI is better than the other PPIs prediction methods for most evaluation metrics, with AUROCs of 82.0%, 78.8%, and 75.5% on the three datasets, respectively, substantially higher than the DELPHI method with 73.7%, 71.1%, and 68.5%, respectively. Furthermore, for the average precision (AP), EDLMPPI beat DELPHI by 12.3%, 9.3%, and 8.1% on the three datasets, respectively, which brings considerable improvement. The reason seems to be that EDLMPPI can address the amino acid long-range dependency problem based on the transformer of self-attentive mechanism, which fully explores the global contextual features and semantic information, indicating that our proposed deep learning architecture provides an important contribution to accurate classification. In addition, we incorporated traditional biological features, such as the evolutionary information and several physicochemical properties, to bridge possible shortcomings of ProtT5, thereby further improving identification performance. Notably, EDLMPPI showed a higher advantage on the Dset_448 dataset, comparing full-length sequences, suggesting that our feature extraction method may be better and more accurate in the functional expression of complete protein sequences. Overall, EDLMPPI has been substantially ahead of existing methods and can be used as a complementary tool for protein–protein interaction site annotation.

### Protein binding domains analysis

Protein domains are closely related to the completion of physiological functions of the proteins and serve as the structural basis for their cellular functions^50^. To gain insight into the potential relationship between protein structural domains and protein–protein interaction sites, we performed an experiment to verify whether EDLMPPI accurately predicts PPIs in the protein domain. We annotated 448 protein sequences in the Dset_448 dataset by Pfam^51^ to remove any overlapping structural domains and finally obtained 501 structural domains. Figure 3b shows the correspondence between structural domains of each size and the number of PPIs in them, while we compare the prediction results of EDLMPPI, DELPHI, and SCRIBER^13^. In addition, we added a control group to enhance the rationality of the experiment: a fragment of the same size as the protein domain was randomly selected from the sequence. From the results, the prediction results of EDLMPPI were more optimistic than the other two methods, with the number of PPIs predicted by EDLMPPI increasing with the growth of the structural domain. According to a previous study^52^, the length-deviant domain superfamilies are highly interacting, more mixed in function, and regulated by multiple proteins, which supports the plausibility of EDLMPPI in predicting protein function. In addition, we counted the proportion of predicted PPIs estimated by EDLMPPI, DELPHI, and SCRIBER for each structural domain and calculated the Pearson correlation coefficient with the true proportion vector. EDLMPPI presented the highest correlation with the native annotations with a score of 0.70, while DELPHI, SCRIBER, and the control group scored 0.63, 0.57, and 0.21, respectively.

To further indicate that EDLMPPI can accurately predict the performance of binding sites in protein domains, we selected three enzyme proteins with high catalytic activity, P19821 - DPO1_THEAQ, P9WHH9 - DLDH_MYCTU, and P17109 - MEND_ECOLI to demonstrate the difference in performance predicted by different methods. Since SCRIBER and DELPHI provided better performance in the prediction of PPIs than other PPI site prediction models, we employed the prediction results of SCRIBER and DELPHI in these three sequence species as comparisons, and the results are displayed in Table 6. With a protein structural domain size of 337 in P19821 - DPO1_THEAQ, the true number of experimentally detected PPIs is 31, and the prediction of EDLMPPI was 36, closer to the true number compared to SCRIBER and DELPHI. This performance is more evident in P9WHH9 - DLDH_MYCTU and P17109 - MEND_ECOLI, where the number of PPIs predicted by EDLMPPI differs from the true value by only 1–2, indicating the effectiveness of EDLMPPI in predicting the binding sites of protein structural domains and also validating our previous conclusion that EDLMPPI can provide more binding sites in the structural domains of proteins.

**Table 6:** Comparison of prediction results of different methods on catalase protein sequences.

	Domin size	Native PPIs	Predicted PPIs
*P19821 - DPO1_THEAQ*			
SCRIBER	337	31	0
DELPHI	337	31	23
EDLMPPI	337	31	36
*P9WHH9 - DLDH_MYCTU*			
SCRIBER	321	37	5
DELPHI	321	37	26
EDLMPPI	321	37	35
*P17109 - MEND_ECOLI*			
SCRIBER	139	38	3
DELPHI	139	38	14
EDLMPPI	139	38	39

### Interpretability analysis

**Fig. 4:**
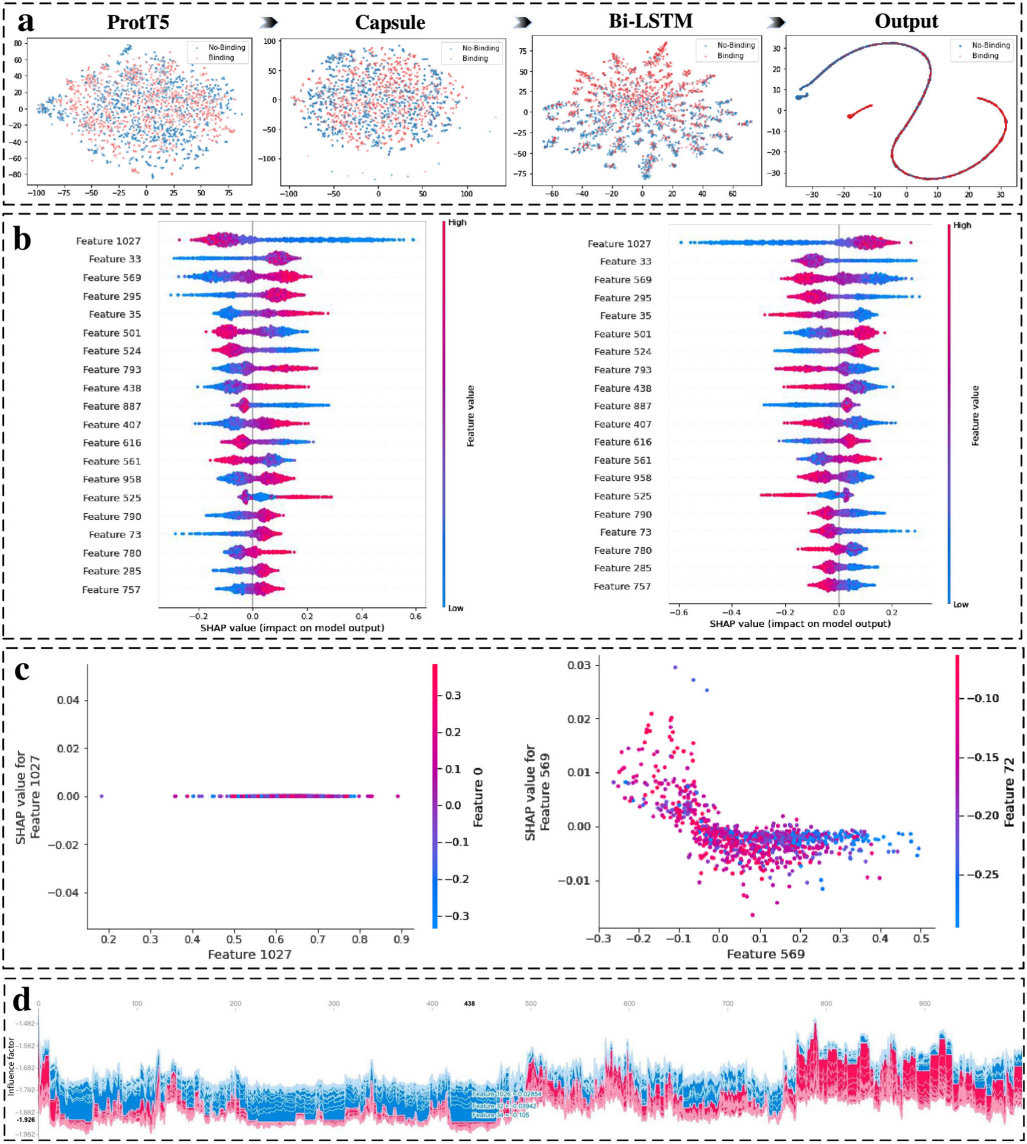
Presentation of the results of the interpretability analysis experiment. **a** The t-SNE flow graph shows the clustering effect of the output of the different intermediate layers of the EDLMPPI architecture. **b** The 20 features that have the greatest impact on PPIs identification, revealing how they act for predicting non-binding sites and bindings sites, respectively. **c** The schematic diagrams show the interaction between feature 1024 and other features, and the interaction between feature 569 and other features, respectively. **d** A stacked diagram showing the effect of each feature on each sample.

To investigate the effectiveness of the EDLMPPI architecture, we extracted the intermediate layer outputs of the model at various stages and mapped them onto a two-dimensional space for clustering, as shown in Fig. 4a. We see that the original embedding was distributed haphazardly, while after the BiLSTM layer, a more obvious clustering effect can be seen. The capsule layer further preserved the key classification features, and the binding and non-binding sites appeared as separate clusters. Finally, after the softmax function, accurate identification was achieved.

In addition, we explored the contributions of different features to the protein–protein binding site recognition and the interaction relationship. Figure 4b shows the 20 features that have the greatest impact on PPIs identification, and reveals how they act in predicting the non-binding sites and bindings sites, respectively. The red color represents higher feature values while the blue represents lower feature values. Taking features 1027 and 33 as examples, the higher feature 1027 tends to classify samples as binding sites while the higher feature 33 is more likely to classify samples as non-binding sites. Compared with the impact of a single feature on the model, the interaction of features was more important. Figure 4c shows how Feature 1027 and Feature 569 interact with the other features. We note that Feature 1027 had no significant interaction with the other features, which is consistent with our judgment that Feature 1027 represents solvent accessibility and is encoded as a vector of length 1, without too much dependency on the other features. On the other hand, a strong correlation was shown between Features 569 and 72, and the effect of Feature 72 on classification was weakened at lower values of Feature 569. This comes from the fact that ProtT5 contains global context dependency, and the expression of features is based on joint action with other features, which further validates the effectiveness of ProtT5. Figure 4d is a stacked diagram showing the effect of each feature on each sample, which allows us to observe which features affect the identification of a sample.

**Fig. 5:**
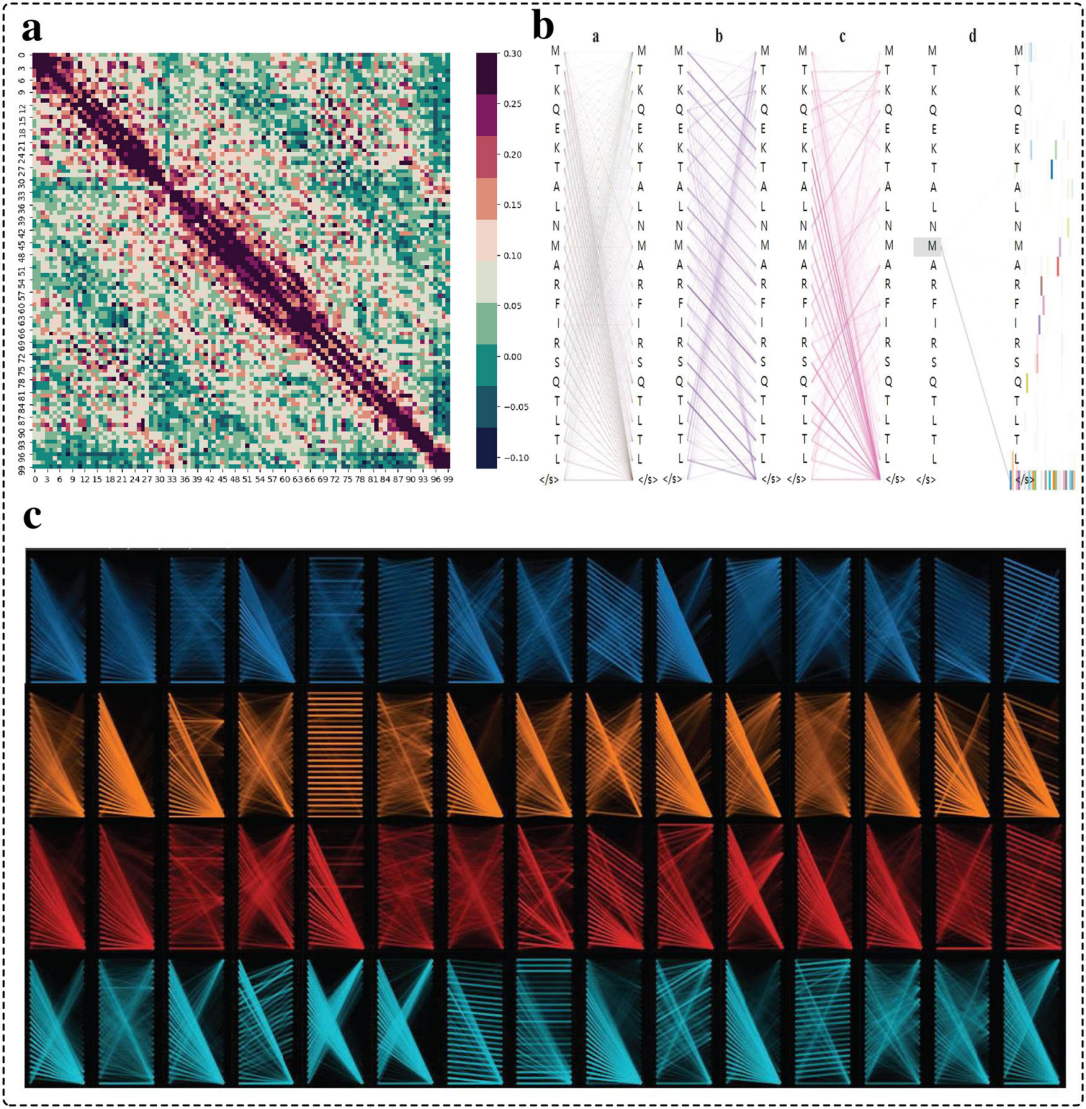
Presentation of the results of the interpretability analysis experiment. **a** Correlation heat map of each residue under ProtT5 embedding. **b** Attention view with different layers and different attention heads. **c** Attention flow view between different layers, with each color representing a different layer.

To gain a deeper understanding of the working of EDLMPPI, we investigated the internal process of ProtT5 embedding for reliability. First, we selected a complete protein sequence and encoded it using ProtT5. For each amino acid embedding vector, we applied the Pearson correlation coefficient to describe the correlation between residues. The results are displayed in Fig. 5a, where we see that each amino acid always had a strong correlation with the amino acid closer to it, but as the distance becomes farther, ProtT5 could still capture an association between amino acids, implying that ProtT5 balanced the local influences and long-term dependence. To further into the process, we applied Bertviz^53^ to visualize each attention head and each layer in ProtT5, and the results are shown in Fig. 5b,c, where the different colors represent the different attention heads and the saturation of the lines represents the attention scores. Figure 5b(a) shows the first layer of attention in all attention heads, which roughly resembles a full connection, implying that for each residue, all attention heads tried to find the association with the target of the other residues. The </*s*> acts as a sequence splitter that carries the attention of all residues, which indicates that for ProtT5, the overall identity of a sequence is determined by all amino acids together. Furthermore, Fig. 5b(d) shows clearly the flow of target amino acids in the different attention heads, verifying our previous statement that higher attention is seen with closer proximity. Moreover, Fig. 5c visualizes the evolution of each attention head in the different layers, as the layers deepened, the attention pattern shifted from focusing on the association between different amino acids to transmitting the expression of the amino acid sequences. In summary, ProtT5 can explore the connection between the protein-level structure and its function from local to global, providing a reasonable interpretation that EDLMPPI effectively predicts protein–protein interaction binding sites.

## The EDLMPPI user interface facilitates exploration of PPIs identification

To facilitate researcher use and improve our model and accelerate progress in protein binding site prediction, we have developed a fully functional EDLMPPI online prediction webserver for PPI, which is available at http://www.edlmppi.top:5002/. Given the limited computational resources and the large computational capacity of ProtT5, we patiently guide users on how to set up the ProtT5 environment in their local environment, download the model, and extract features in various scenarios. Furthermore, the online prediction server also encourages users to upload the extracted ProtT5 features directly on the EDLMPPI server, so that we can return the prediction results via email with an interpretation of the results. In addition, we have synchronized open source data and code on GitHub, which can be accessed at https://github.com/houzl3416/EDLMPPI.git.

Supplementary Figure 1 summarizes the main modules and Supplementary Figure 1a illustrates the main interface, which includes three ways of obtaining ProtT5: extracting it onto your device, extracting it onto Colab, or downloading the file we provide. Supplementary Figure 1b shows the prediction module: once the ProtT5 features are uploaded, the server can send the prediction results to the user’s email automatically. Finally, there is the downloadable module depicted in Supplementary Figure 1c, where users can directly click on the links to download datasets and models in a rapid manner.

## Conclusions

In this study, we propose a protein–protein interaction site prediction method based on ensemble deep learning models, called EDLMPPI, EDLMPPI adapts the dynamic word embedding model based on transformer architecture to the study of protein–protein interaction sites and uses ProtT5 to capture the contextual and positional information between residues, while integrating eleven multi-source biological features to further enrich the feature representation. Meanwhile, we developed a multi-channel integrated deep learning model that captures both local context dependence and global context dependence of protein sequences and effectively solves the data imbalance problem.

To demonstrate the effectiveness of the EDLMPPI, we compared it to ten different traditional machine learning and deep learning models on three widely-used benchmark datasets. Moreover, we compared EDLMPPI with other PPI website prediction models and the predictive performance of EDLMPPI improves prediction over these models. Besides, in the prediction of PPIs in protein structural domains, EDLMPPI shows more biologically consistent results, which indicates that EDLMPPI has the ability for certain biological analysis and can be used to guide biologists to make specific experiments on proteins. Meanwhile, the interpretability analysis fully demonstrates the internal vision of the EDLMPPI model, which further enhances the rationality of the model.

In addition, the release of the EDLMPPI online prediction web server provides detailed guidance on model training and prediction, ensuring that the results of our experiments are repeatable and operational. The code and data are also open-sourced at https://github.com/houzl3416/EDLMPPI.git.

In summary, EDLMPPI is a very competitive protein–protein interaction site prediction tool with the advantages of high efficiency and accuracy, proving a new alternative for protein interaction site identification. It provides new ideas and insights into the task of protein–protein interaction site prediction and can also serve as an important assistant for biologists to effectively implement PPI prediction and downstream analysis work. The release of the webserver also greatly facilitates the work of other researchers to improve our model and achieve more effective prediction results. In the future, we will incorporate other dynamic word embedding models into our proposed model and adapt them to other relevant protein identification problems.

## Methods

### Datasets

For datasets, we collected three widely-used benchmark datasets, Dset_186^54^, Dset_72^54^, and Dset_164^55^. Dset_186 was constructed from the PDB database^3^ and contains 186 protein sequences with a resolution of <3.0 Å and sequence homology <25%. This dataset was refined in multiple steps, including the removal of chains with identical UniprotKB/Swiss-Prot accessions, the removal of transmembrane proteins, the removal of dimeric structures, the removal of proteins with surface accessibility and interfacial polarity buried within a certain range, and the removal of similarities. Dset_72 and Dset_164 were constructed in the same manner as Dset_186, and consist of 72 and 186 protein sequences, respectively.

Further Dset_1291 is a dataset from the BioLip database, where a binding site is defined if the distance between an atom of a residue and an atom of a given protein partner is 0.5 Å plus the sum of the van der Waals radii of the two atoms^13^. Zhang et al.^13^ eliminated the fragmented proteins and then transferred the annotation of the bound residues to the same UniProt sequence. Therefore, the similarity between the sequences was reduced to less than 25% under the Blast-Clust method. Finally, Dset_843 (843 sequences of Dset_1291) was used to train our model, while the remaining 448 sequences (Dset_448) were employed as the independent test set.

Using these datasets, we constructed the training and test sets. As Dset_843 and Dset_448 consist entirely of full-length protein sequences, while Dset_71, Dset_186, and Dset_164 are composed of fragmented sequences; to enhance the generalizability of the model, we selected Dset_843 and Dset_186 representing two different types of datasets as our training datasets, respectively. Then Dset_448, Dset_72, and Dset_164 were used as independent test sets to test the performance of the different PPI site prediction models. In addition, to reduce the similarity between the training and test sets, we performed consistency redundancy removal between them using the PSI-BlAST^56^ procedure to ensure the similarity was below 25%. Supplementary Table 1 summarizes the number of protein residues and the proportion of binding sites in each dataset, where it is easy to see that the distribution of the datasets is relatively unbalanced, with positive samples accounting for only 10–18% of the total sample size, which poses a challenge for the generalizability of the model.

### Feature descriptors

To fully explore the structural characteristics of protein–protein interaction sites, several features, including dynamic global contextual information and multi-source biological features, are extracted from protein sequences as follows.

#### Dynamic global contextual information

Due to the expensive cost of traditional biological experiments and the low capability of some deep learning-based techniques, we introduce the dynamic word embedding-based ProtT5^24^ to represent the feature expression information of proteins to obtain the global context-sensitive information between the different sequences and amino acids, which has already been proven to be an effective method experimentally. Specifically, ProtT5 is employed for generating global contextual embeddings. Indeed, ProtT5 learns a positional encoding for each attention head in the transformer architecture and shares it on all levels. In ProtT5, the training corpus is Uniref50, which contains 45 million protein sequences composed of 15 billion amino acids. Such a huge training set guarantees that ProtT5 will capture the structural and functional connections between different types or races of proteins.

ProtT5 first maps each amino acid into a fixed-length vector by means of an embedding layer, besides, the position embedding in ProtT5 is employed to encode the relative positional information of each amino acid in the corresponding protein sequence, and the segment embedding was introduced to distinguish the different protein sequences. The sum of the token embedding, segmentation embedding, and position embedding provides not only a non-contextual mapping of amino acids to the underlying space but also extends the amino acid dependencies in each protein sequence and the contextual associations between different protein sequences, which can be defined as follows:

1 $${E}_{word}	=\, {E}_{tok}+{E}_{seg}+{E}_{pos}\\ 	=\, {O}_{tok}{W}_{tok}+{O}_{seg}{W}_{seg}+{O}_{pos}{W}_{pos}$$

where *W*_*tok*_, *W*_*seg*_, and *W*_*pos*_ are the corresponding parameter matrices to be trained. After that, dynamic word embedding, learned from the multi-head self-attention mechanism in the transformer architecture, is used to correlate the relevant amino acids in the protein sequence, which can be calculated through the following formula:

2 $$X{W}_{i}^{Q}={Q}_{i},\quad X{W}_{i}^{K}={K}_{i},\quad X{W}_{i}^{V}={V}_{i},\quad i=1,\ldots ,m$$

3 $${Z}_{i} 	 =\, {{{{{{{\rm{Attention}}}}}}}}{\left({Q}_{i},{K}_{i},{V}_{i}\right)}_{i}\\ 	 =\, {{{{{{{\rm{SoftMax}}}}}}}}\left(\frac{{Q}_{i}{K}_{i}^{T}}{\sqrt{{d}_{k}}}\right){V}_{i},\quad i=1,\ldots ,m$$

4 $${{{{{{{\rm{MultiHead}}}}}}}}(Q,K,V)={{{{{{{\rm{Concat}}}}}}}}\left({Z}_{1},\ldots ,{Z}_{m}\right){W}^{O}$$

where *Q*(*Query*), *K*(*Key*), *V*(*Value*) are obtained through *m* linear transformations, which are used to store all word embeddings. *Z*_*i*_ represents the attention of each attention head, which is calculated by the linear transformation of a set of *Q*, *K*, *V*.

Indeed, the attention stack of ProtT5 consists of 24 layers, each layer contains 32 attention heads, and the size of the hidden layer is 1024. This stacked mode is what allows each layer to operate on the output of the previous layer. Through such a repeated combination of word embedding, ProtT5 can form a very rich representation as it reaches the deepest layer of the model^23^. Therefore, in our study, we extract the embedding of the last layer of the attention stack into our feature representation.

#### Multi-source biological features

Further, to improve the prediction performance, we accessed the evolutionary information, physical properties, and physicochemical properties of protein residues to enrich the feature expression.

*(1) Position-Specific Scoring Matrix (PSSM):* PSSM provides a flexible way to represent the specificity of residue interactions, which describes the evolutionary conservation of the residue positions. It can be described as follows:

5 $${{{{{{{\rm{score}}}}}}}}(a,b)={\log }_{10}\left(M(a,b)/{p}_{a}{p}_{b}\right)$$

where *p*_*a*_ and *p*_*b*_ represent the probability of observing amino acids *a* and *b*, respectively, and *M*(*a*, *b*) is the probability score of a mutation. We chose Uniref90 as the comparison database, set the number of iterations to three, and set the threshold value to 0.001 by PSI-BLAST.

*(2) Physical characteristics:* Physical characteristics are the graph index, polarization rate, normalized van der Waals volume, hydrophobicity, isoelectric point, spiral probability, and sheet probability. The same calculations are performed using the values reported in ref. ^57^ to obtain a 7-dimensional vector for each amino acid.

*(3) Physicochemical properties:* To accurately express the differences and connections between different residues, we introduce the physicochemical properties of amino acids. The physicochemical characteristics of a residue are described by three values: the number of atoms, the number of electrostatic charges, and the number of potential hydrogen bonds. These values are only related to the type of amino acid and do not contain any structural information from the amino acid residue.

### Ensemble deep memory capsule network

To capture the crucial information in the hybrid feature schemes more efficiently, we developed the ensemble deep memory capsule network (EDMCN) to maximize the feature learning performance of protein–protein interaction site identification, as depicted in Fig. 1. Deep memory capsule networks expand the parallelism of traditional memory networks by linking them with different output sizes to capture the correlation between amino acids at different depth scales. Besides, the capsule structure can further explore the intrinsic connections between features and retain location information between samples. In addition, to promote the generalization and stability of the model, we introduced an asymmetric bagging algorithm to solve the high imbalance between samples.

#### Deep memory network

Traditional memory networks such as LSTM^39^, GRU^40^, etc. have achieved good results in organizing the context of features for prediction. However, these models are parameter-sensitive, which greatly affects the stability of the prediction. To address this, we developed a deep memory network to enhance the generalization performance of the model. The central idea of deep memory networks is to connect multiple memory networks with different output scales to capture the correlation between residues in a multi-scale manner. Formally, it mainly controls the protein information flow through three gates (input gate(i), forget gate(f), and output gate(o)), including when to remember, update, and utilize the information. The forget gate works by accepting a long-term memory *M*_*t*−1_ and deciding on which parts to retain or discard. In a time step *t*, the forget gate first calculates the forgetting factor *f*_*t*_ from the previous hidden state *h*_*t*−1_ and the current input information *m*_*t*_:

6 $${f}_{t}=\sigma \left({W}_{f}\cdot \left[{h}_{t-1},{m}_{t}\right]+{b}_{f}\right)$$

where *σ* is the logistic sigmoid function. The input gate mainly controls which input currents *m*_*t*_ can pass through the memory cell, first by generating a control signal to control the rate *r*_*t*_ of inflow:

7 $${r}_{t}=\sigma \left({W}_{r}\cdot \left[{h}_{t-1},{m}_{t}\right]+{b}_{r}\right)$$

Next, the input gate generates candidate memory cells $\widetilde{{M}_{t}}$ and calculates the memory information that eventually passes through the input gate based on the previously solved *r*_*t*_:

8 $${\widetilde{M}}_{t}=\tanh \left({W}_{M}\cdot \left[{h}_{t-1},{m}_{t}\right]+{b}_{M}\right)$$

9 $${M}_{t}={f}_{t}* {M}_{t-1}+{r}_{t}* {\widetilde{M}}_{t}$$

Finally, the output gate filters *m*_*t*_ by generating the control signal *g*_*t*_ to obtain the output *O*_*t*_:

10 $${g}_{t}=\sigma \left({W}_{g}\cdot \left[{h}_{t-1},{m}_{t}\right]+{b}_{g}\right)$$

11 $${O}_{t}={g}_{t}* \tanh \left({M}_{t}\right)$$

#### Capsule network

Deep memory network effectively captures global contextual dependencies among features, however, it tends to weaken the strong correlations among local features and lose topological information about feature types. To solve this problem, we introduce the capsule network^27^. Intuitively, the capsule network contains a convolutional network part along with neurons called capsules, which decide its perception of features, not only reflected in the importance of the features but also the various states of the features, including their location information. In this way, the capsule network can effectively capture the potential associations between features for our highly context-dependent feature description methods.

The structure of capsule neurons in a capsule network is shown in Fig. 1. In a capsule network, the capsule neurons are connected in a similar way as a full connection, for the current layer of capsules *c*_1_, *c*_2_, …, *c*_*i*_, the position relationship between the local and global features is learned through the pose transformation (translation, rotation, deflation):

12 $${\hat{c}}_{j| i}={W}_{ij}{c}_{i}$$

where *W*_*ij*_ is the weight matrix. Then, we multiply each transformed vector by a coupling coefficient *o*_*ij*_ and pass it to the next layer of capsules, and sum all the neuron signals received by the *j*-th capsule of the next layer:

13 $${s}_{j}=\mathop{\sum}\limits_{i}{o}_{ij}{\hat{c}}_{j| i}$$

and the *o*_*ij*_ can be calculated as follows:

14 $${o}_{ij}=\frac{{{{{{{{{\rm{e}}}}}}}}}^{{b}_{ij}}}{{\sum }_{n}{{{{{{{{\rm{e}}}}}}}}}^{{b}_{in}}}$$

where *b*_*ij*_ is the logarithmic prior probability of whether two capsules are connected. Similar to sigmoid, a nonlinear activation function called squash^27^ is employed for mapping vectors to [0, 1], and the capsule output *v*_*j*_ of this layer can be calculated as follows:

15 $${v}_{j}=\frac{{\left\Vert {s}_{j}\right\Vert }^{2}}{1+{\left\Vert {s}_{j}\right\Vert }^{2}}\frac{{s}_{j}}{\left\Vert {s}_{j}\right\Vert }$$

#### Ensemble deep learning algorithm

To further improve the stability and generalization performance of our proposed model, an ensemble learning method based on the asymmetric bagging algorithm^58^ is applied to deal with the skewed distribution of categories in unbalanced datasets. Bagging is one of the prevailing ensemble learning methods^59^, which can integrate the prediction results of multiple different classifiers and then use the voting principle to determine the class of the samples in the decision phase, aiming to reduce variance and promote the generalization performance of the model. Indeed, the principle of variance reduction by bagging is represented by the following equation:

16 $${{{{{{{\rm{Var}}}}}}}}(cX)	=\, E\left[{(cX-E[cX])}^{2}\right]\\ 	=\, {c}^{2}E\left[{(X-E[X])}^{2}\right]\\ 	=\, {c}^{2}{{{{{{{\rm{Var}}}}}}}}(X)$$

17 $${{{{{{{\rm{Var}}}}}}}}\left({X}_{1}+\cdots +{X}_{n}\right)={{{{{{{\rm{Var}}}}}}}}\left({X}_{1}\right)+\cdots +{{{{{{{\rm{Var}}}}}}}}\left({X}_{n}\right)$$

18 $${{{{{{{\rm{Var}}}}}}}}\left(\frac{1}{n}\mathop{\sum }\limits_{i=1}^{n}{X}_{i}\right)=\frac{1}{{n}^{2}}{{{{{{{\rm{Var}}}}}}}}\left(\mathop{\sum }\limits_{i=1}^{n}{X}_{i}\right)=\frac{{\sigma }^{2}}{n}$$

where *X* represents an independent sample, *Var*(*X*) is the variance, and *E*(*X*) represents the mean of sample *X*. Then, it can be seen that assuming there are *n* independent models with an identical distribution and the variance of each model is *σ*^2^, the variance of the ensemble model can be deduced from Eqs. (16) and (17) as *σ*^2^/*n*. Bagging is sampled with put-back sampling so that there are duplicate samples between data sets, thus violating the independence assumption in Eq. (18). In this case, the variance of the ensemble model based on the correlation coefficient *rho* between the individual models can be expressed as follows:

19 $${{{{{{{\rm{Var}}}}}}}}\left(\frac{1}{n}\mathop{\sum }\limits_{i=1}^{n}{X}_{i}\right)=\frac{{\sigma }^{2}}{n}+\frac{n-1}{n}\rho {\sigma }^{2}$$

Under that, as the number of classifiers increases or the correlation between single models decreases, the variance of the ensemble model further decreases. Motivated by the above observations, we proposed to employ the asymmetric bagging algorithm to achieve this goal. For the dataset *S*, in each iteration, we keep all the samples of protein binding sites as *S*_*p*_, and separate a subset ${S}_{n}^{{\prime} }$ with the same scale as *S*_*p*_ from the samples *S*_*n*_ of non-binding sites. This step is repeated for sampling without replacement until the training process covers all samples, and eventually, multiple classifiers can be obtained. After that, we sum the softmax values obtained by these multiple classifiers for each sample to make the final identification decision. On this basis, asymmetric bagging can adequately ensure a balanced class distribution of the input data for each model and keep the correlation between individual models as low as possible. It is worth mentioning that although the ensemble models may increase the computational complexity, the feasibility of parallelism in asymmetric bagging can effectively reduce the running time with sufficient computational resources.

### Parameter settings

To demonstrate the effectiveness of our proposed EDLMPPI, we compare it to several traditional machine learning methods and deep learning methods. In the following section, we present the details of the parameter settings of these algorithms.

#### Deep learning algorithms

For EDLMPPI, we use the tanh function as the activation function and adopt the Glorot initializer with a uniform distribution to initialize the weights for the BiLSTM part. Then, for the number of neurons in the hidden layer, we fix a set of candidate values [32, 64, 128, 256]. For the capsule network, the main hyperparameters are the number of neural capsules and the dimensionality of each neuronal vector, for which we set a group of candidate values [32, 64, 128, 256] and [3, 5, 7, 10], respectively. To obtain the best hyperparameters, we optimize the three sets of candidate values above by the grid search method under Tensorflow 2.5.0 and Keras 2.4.3. The epochs are set to 100 and the early stop mechanism is applied to prevent overfitting of the proposed algorithm.

To conduct a fair comparison to the other deep learning algorithms including TextCNN^38^, Single-Capsule^27^, BiLSTM^39^, BiGRU^40^, and MultiHead Attention^41^, to conduct a fair comparison, the hyperparameter optimization methods used the same principles as EDLMPPI; we also adopted the same rules of the hyperparameter optimization method as for EDLMPPI, using a grid search procedure to select reasonable hyperparameters. For TextCNN, the test settings for different combinations of convolutional kernels of different sizes were {{1, 3, 5, 7}, {7, 9, 11, 13}, {4, 5, 6, 7}, {7, 8, 9, 10}}, where the number of filters for each combination is chosen from 16, 32, 64, 128, respectively. The number of hidden layer cells of BiLSTM and BiGRU is chosen from {32, 64, 128}. In the capsule network, the candidate values for the number of neural capsules and the dimensionality of each neuronal vector are {32, 64, 128, 256} and {3, 5, 7, 10}, respectively. Finally, the Multi-Head attention network selects the number of attention heads from {4, 8, 16, 32}.

#### Machine learning algorithms

The machine learning methods contain three ensemble learning methods (XGBoost^35^, LightGBM^36^, and CatBoost^37^), SGDClassifier (Stochastic Gradient Descent), and MLPClassifier (Multi-Layer Perceptron), which are under the scikit-learn^60^ dependency package in Python environment. XGBoost adopts a level-wise decision tree construction strategy, LightGBM uses a leaf-wise construction strategy and CatBoost applies a symmetric tree structure with full binary decision trees. The SGDClassifier is a stochastic gradient descent learning model with a regularized linear method. The loss gradient is estimated for each sample at a time, and the model is updated in the process using an intensity-decreasing schedule. MLP is a forward-structured artificial neural network, which can solve complex problems quickly. The grid search procedure is also performed to find the optimal hyperparameters for these five classifiers. The candidate parameters and the optimal parameter combinations are summarized in Supplementary Table 2.

### Evaluation performance

To evaluate the performance of different computational methods, we used sensitivity (TPR), specificity (TNR), precision (Pre), accuracy (ACC), F1-score (F1), the Matthews correlation coefficient (MCC), the area under the receiver operating characteristic curve (AUROC), and average precision (AP) as measurement criteria, which can be formulated as below:

20 $${{{{{{{\rm{TPR}}}}}}}}=\frac{{{{{{{{\rm{TP}}}}}}}}}{{{{{{{{\rm{TP}}}}}}}}+{{{{{{{\rm{FN}}}}}}}}}$$

21 $${{{{{{{\rm{TNR}}}}}}}}=\frac{{{{{{{{\rm{TN}}}}}}}}}{{{{{{{{\rm{TN}}}}}}}}+{{{{{{{\rm{FP}}}}}}}}}$$

22 $${{{{{{{\rm{Pre}}}}}}}}=\frac{{{{{{{{\rm{TP}}}}}}}}}{{{{{{{{\rm{TP}}}}}}}}+{{{{{{{\rm{FP}}}}}}}}}$$

23 $${{{{{{{\rm{ACC}}}}}}}}=\frac{{{{{{{{\rm{TP}}}}}}}}+{{{{{{{\rm{TN}}}}}}}}}{{{{{{{{\rm{TP}}}}}}}}+{{{{{{{\rm{FN}}}}}}}}+{{{{{{{\rm{TN}}}}}}}}+{{{{{{{\rm{FP}}}}}}}}}$$

24 $${{{{{{{\rm{F}}}}}}}}1=2\times \frac{{{{{{{{\rm{TPR}}}}}}}}\times {{{{{{{\rm{Pre}}}}}}}}}{{{{{{{{\rm{TPR}}}}}}}}+{{{{{{{\rm{Pre}}}}}}}}}$$

25 $${{{{{{{\rm{MCC}}}}}}}}=\frac{{{{{{{{\rm{TP}}}}}}}}\times {{{{{{{\rm{TN}}}}}}}}-{{{{{{{\rm{FN}}}}}}}}\times {{{{{{{\rm{FP}}}}}}}}}{\sqrt{({{{{{{{\rm{TP}}}}}}}}+{{{{{{{\rm{FP}}}}}}}})\times ({{{{{{{\rm{TP}}}}}}}}+{{{{{{{\rm{FN}}}}}}}})\times ({{{{{{{\rm{TN}}}}}}}}+{{{{{{{\rm{FP}}}}}}}})\times ({{{{{{{\rm{TN}}}}}}}}+{{{{{{{\rm{FN}}}}}}}})}}$$

where true positives (TP) and false positives (FP) represent the number of correctly-predicted binding sites and incorrectly predicted binding sites, respectively. True negatives (TN) and false negatives (FN) represent the number of correctly predicted non-binding sites and incorrectly-predicted non-binding sites, respectively. TPR describes the proportion of correctly predicted binding sites in all positive samples, TNR indicates the proportion of correctly predicted non-binding sites in the total negative samples, and Pre represents the probability of correct prediction in all samples with predicted binding sites.

In unbalanced data, since ACC cannot accurately capture the strengths of the model, we adopted ACC as an additional metric for evaluation. In addition, another two metrics, AUROC and AP are calculated related to the predicted probability of each amino acid to measure the unbalanced data. AUROC is not influenced by sample imbalance and can accurately measure model performance in unbalanced data^61^. AP is a weighted average of the accuracy of each threshold in the dataset, with the change in recall as the weight, which can be defined as follows:

26 $${{{{{{{\rm{AP}}}}}}}}=\mathop{\sum}\limits_{n}\left({R}_{n}-{R}_{n-1}\right){P}_{n}$$

where *R*_*n*_ and *P*_*n*_ are the recall and precision at the *n*-th threshold.

### Statistics and reproducibility

The statistical analyses of the data were conducted using the Python software package. We used the asymmetric bagging algorithm to focus on the imbalance of the data to reduce its impact on the experimental results. The reproducibility of experiments was ensured by performing a minimum of three independent replicates for each condition. Replicates were performed by different researchers, and the data were combined and analyzed using appropriate statistical tests. Overall, our experiments were designed to be highly reproducible. All materials and procedures were clearly described in the methods section, and the data were carefully collected and analyzed using standard statistical methods. We believe that these measures have increased the reliability and reproducibility of our results.

### Reporting summary

Further information on research design is available in the Nature Portfolio Reporting Summary linked to this article.

## Supplementary information

Supplementary Information

Reporting Summary

## Data Availability

We collected four widely-used benchmark datasets, Dset_186, Dset_72, Dset_164, and Dset_1291. Dset_186, Dset_72, and Dset_164 were constructed from the PDB database and contains 422 protein sequences with a resolution of <3.0 Å and sequence homology <25%. Dset_1291 is a dataset from the BioLip database, where a binding site is defined if the distance between an atom of a residue and an atom of a given protein partner is 0.5 Å plus the sum of the van der Waals radii of the two atoms. All data sets are available for download at http://www.edlmppi.top:5002/ or https://github.com/houzl3416/EDLMPPI.git. Besides, the numerical source data for graphs and charts can be downloaded at 10.6084/m9.figshare.21778913.v1.

## Code availability

All the code is available at https://github.com/houzl3416/EDLMPPI.git.
