# Supplementary material for: Learning the protein language of proteome-wide protein-protein binding sites via explainable ensemble deep learning
Source: Commun Biol. 2023 Jan 19;6:73. doi: 10.1038/s42003-023-04462-5 (PMC9849350; doi:10.1038/s42003-023-04462-5)
Supplement: Supplementary file 1 — Supplementary Information [file 42003_2023_4462_MOESM1_ESM.pdf]

Supplementary Material

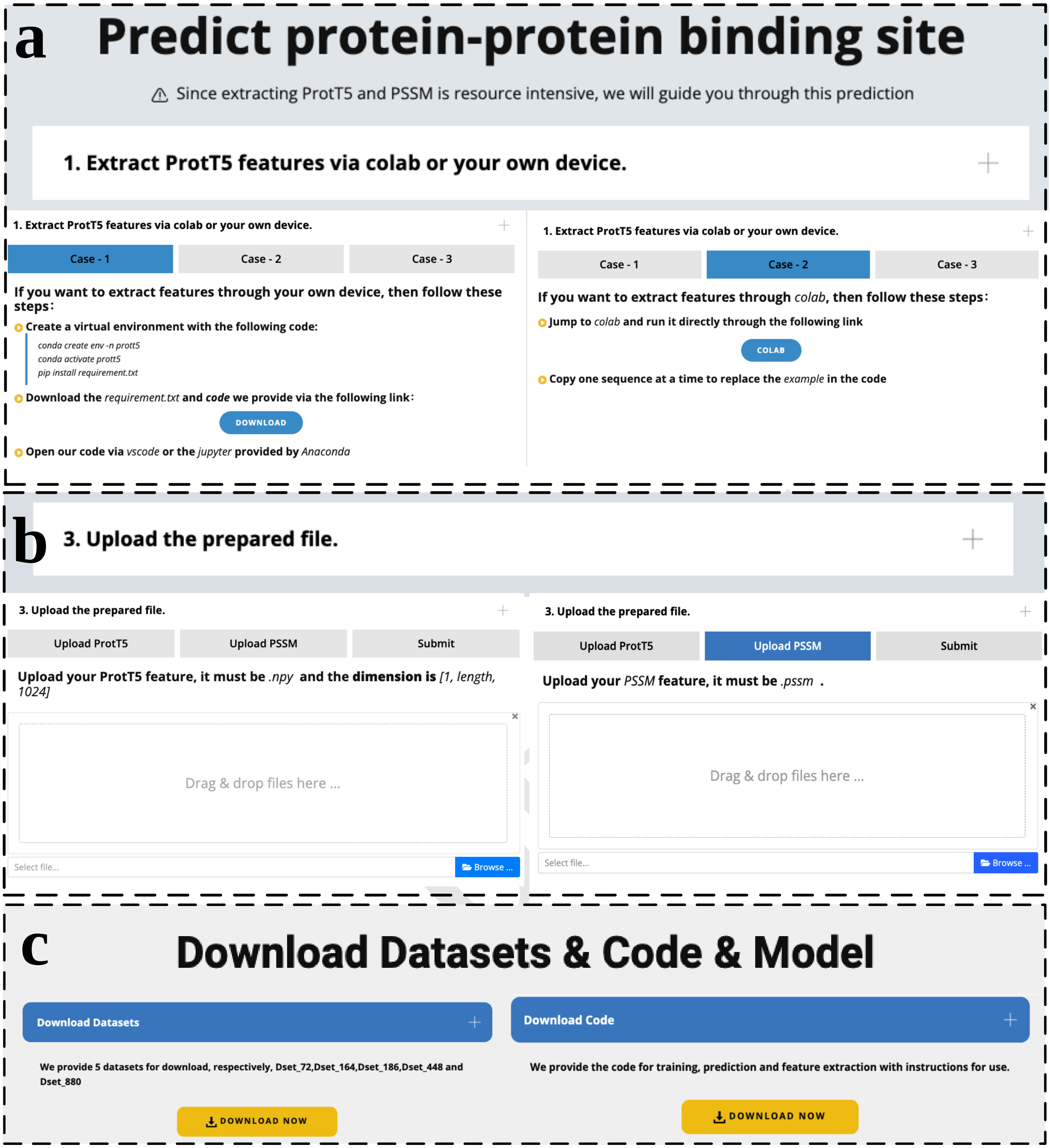

Supplementary Figure 1 Schematic diagram of web server functional modules. (A) Guidance Control: Guides users for extracting ProtT5 in defined situations. (B) Submit Form: The user can submit ProtT5 and PSSM for the server to complete the prediction. (C) Download Module: Provides high-speed download links for data sets, code, and pre-trained models.

Supplementary Table 1. Details of the training and test sets of our study.

	Dataset	ResiduesNumber	Binding Ratio %
Train	Dset_843	450 598	10.14
	Dset_186	36 219	15.23
Test	Dset_72	18 140	10.60
	Dset_164	33 681	18.10
	Dset_448	116 500	13.57

Supplementary Table 2. Hyperparameter candidates and optimal parameter combinations for the traditional machine learning algorithms.

Algorithms	Parameter Candidate Values	Optimal parameters
XGBoost	{'max_depth': [3, 4, 5, 6, 7, 8], 'eta':[0.001, 0.01, 0.1], 'gamma':[0, 0.001, 0.01], 'n_estimators':[100, 500, 1000]}	[4, 0.01,0, 500]
CatBoost	{'depth': [5, 6, 7],'learning_rate' : [0.01, 0.03, 0.1], 'l2_leaf_reg': [1, 4, 9], 'iterations': [100, 300, 1000]}	[7, 0.1, 9, 300]
LGBM	{'n_estimators':range(100, 500, 100), 'learning_rate':[0.01, 0.05, 0.1], 'feature_fraction':[0.7, 1]}	[400, 0.1, 0.7]
SGD	{'loss':['log', 'modified_huber'], 'penalty':['l2', 'l1', 'elasticnet'], 'alpha':[0.0001, 0.001, 0.01]}	['huber', 'elasticnet','0.001']
MLP	{'solver':['lbfgs', 'sgd', 'adam'], 'alpha':[1e-5, 1e-4, 0.001, 0.01]}	['lbfgs', 1e-5]
